# Identifying diagnosis evidence of cardiogenic stroke from Chinese echocardiograph reports

**DOI:** 10.1186/s12911-020-1106-3

**Published:** 2020-07-09

**Authors:** Lu Qin, Xiaowei Xu, Lingling Ding, Zixiao Li, Jiao Li

**Affiliations:** 1 Institute of Medical Information, Chinese Academy of Medical Sciences/ Peking Union Medical College, Beijing, China; 2 Beijing Tiantan Hospital, Capital Medical University, Beijing, China

**Keywords:** Cardiogenic stroke, Diagnosis evidences, Chinese echocardiograph reports, BiLSTM-CRF

## Abstract

**Background:**

Cardiogenic stroke has increasing morbidity in China and brought economic burden to patient families. In cardiogenic stroke diagnosis, echocardiograph examination is one of the most important examinations. Sonographers will investigate patients’ heart via echocardiograph, and describe them in the echocardiograph reports. In this study, we developed a machine learning model to automatically identify diagnosis evidences of cardiogenic stroke providing to neurologist for clinical decision making.

**Methods:**

We collected 4188 Chinese echocardiograph reports of 4018 patients, with average length 177 Chinese characters in free-text style. Collaborating with neurologists and sonographers, we summarized 149 phrases on diagnosis evidence of cardiogenic stroke such as “二尖瓣重度狭窄” (severe mitral stenosis), “主动脉瓣退行性变” (aortic valve degeneration) and so on. Furthermore, we developed an annotated corpus via mapping 149 phrases to the 4188 reports. We selected 11 most frequent diagnosis evidence types such as “二尖瓣狭窄” (mitral stenosis) for further identifying. The generated corpus is divided into training set and testing set in the ratio of 8:2, which is used to train and validate a machine learning model to identify the evidence of cardiogenic stroke using BiLSTM-CRF algorithm.

**Results:**

Our machine learning method achieved the average performance on the diagnosis evidence identification is 98.03, 90.17 and 93.94% respectively. In addition, our method is capable to identify the novel diagnosis evidence of cardiogenic stroke description such as “二尖瓣中-重度狭窄” (mitral stenosis), “主动脉瓣退行性病变” (aortic valve calcification) et al.

**Conclusions:**

In this study, we analyze the structure of the echocardiograph reports and summarized 149 phrases on diagnosis evidence of cardiogenic stroke. We use the phrases to generate an annotated corpus automatically, which greatly reduces the cost of manual annotation. The model trained based on the corpus also has a good performance on the testing set. The method of automatically identifying diagnosis evidence of cardiogenic stroke proposed in this study will be further refined in the practice.

## Background

Ischemic stroke is the most common type of stroke, which accounts for 69.6 to 70.8% of stroke in China [1, 2]. With the continuous acceleration of the aging population and urbanization, the unhealthy lifestyle of residents is becoming popular, which results in the sharply rising incidence of stroke and brings heavy burden on families and societies in China [3, 4]. The National Health Commission of the Peoples’ Republic of China has adopted a series of policies and methods for stoke prevention and control [5, 6]. The accurate classification of ischemic stroke has significant impact on the treatment of patients [7] and stroke-related studies such as clinical trial [8], epidemiology [9] and gene study [10]. Referring to the international stroke classification, Gao et al. proposed the Chinese Ischemic Stroke Subclassification (CISS) [11], which is suitable for stroke classification in China. In CISS the Ischemic stroke is divided into five categories: large artery atherosclerosis (LAA), cardiogenic stroke (CS), penetrating artery disease (PAD), other etiology (OE) and undetermined etiology (UE). Among them, cardiogenic stroke is one of the most common type of acute ischemic stroke, which is caused by a variety of cardiac sources of embolism and accounts for 20% of stroke [12]. Cardiac sources of embolism are diagnosis evidences of cardiogenic stroke. In the clinical practice, neurologists make decisions mainly depending on the interpretation of echocardiography reports and electrocardiogram reports. The echocardiography reports reflect the cardiac sources of embolism related to the abnormal structure and function of the heart, while the electrocardiogram reports reflect the cardiac sources of embolism related to abnormal cardiac rhythm [13]. The automatic identification of cardiac sources of embolism from the echocardiography reports will lighten the burden of neurologists at a certain extent, and it will also reduce the erroneous diagnosis caused by the misinterpretation of the reports.

This study aims to automatically identify diagnosis evidences of cardiogenic stroke (cardiac sources of embolism) from echocardiography reports to provide to neurologists for clinical decision making. Through consulting clinicians and analyzing thousands of echocardiography reports, we define the task is to identify diagnosis evidences of cardiogenic stroke from the description and conclusion part of echocardiography reports using named entity recognition (NER) technologies.

### Related work

In recent years, computer technology, especially deep learning, has been widely used in medical field, such as clinical diagnosis, treatment, health management, hospital administration and management [14]. Ultrasound examination, as a noninvasive, painless, convenient and intuitive examination, has been widely used in clinical practice [15]. At present, research in ultrasound are mainly focused on ultrasound images and ultrasound reports. The research on ultrasound images belongs to computer vision research, including ultrasound image-based tumor identification, cardiac cycle identification and so on [16, 17]. While the research on ultrasound reports belongs to computer text research, including examination recommendations, ultrasound reports structuration using natural language processing (NLP) technologies and so on [18, 19]. There are also cross study in ultrasound images and reports. Zeng et al. [20] used visual geometry group 16 network (VGG16Net) model to extract features from ultrasound image, and generated description text of ultrasound image automatically, which was helpful for sonographers to understand the content of ultrasound images more quickly and conveniently.

Name entity recognition (NER) is a natural language processing technology to identify target entities from narrative, which has been widely used in the process of ultrasound report. Many machine learning algorithms have been applied to improve the accuracy of NER. Miao [21, 22] et al. compared the performance of rule-based, conditional random field (CRF) and recurrent neural network (RNN) methods in extracting 21 kinds of entities from breast ultrasound reports, in which the F1 score of the three methods on the testing set were 0.85, 0.88 and 0.90 respectively. The results showed that the RNN model had the best performance. Chen [23] et al. used CRF model to extract seven kinds of entities in vascular ultrasound reports of head and neck automatically, whose results were used to generate intelligent treatment suggestions for further treatment of cerebrovascular diseases. Meanwhile, how to improve the efficiency and accuracy of annotation during the process of NER model construction is also a hot research topic. Komiya [24] et al. compared the methods of semi-automatic annotation (revise the result of NER model annotation) and manual annotation, which showed that the semi-automatic was faster and achieved better performance. At the same time, the research also pointed out that manual annotation should be used when there were great differences between the corpus to be annotated and the corpus training the NER model. At present, there is no research on extracting the diagnosis evidences of cardiogenic stroke from echocardiography reports. In this study, an automatic method is explored to annotate echocardiography reports, and then the identifying diagnosis evidences of cardiogenic stroke model will be constructed based on the annotated corpus.

## Methods

### Workflow of the research

**Fig. 1:**
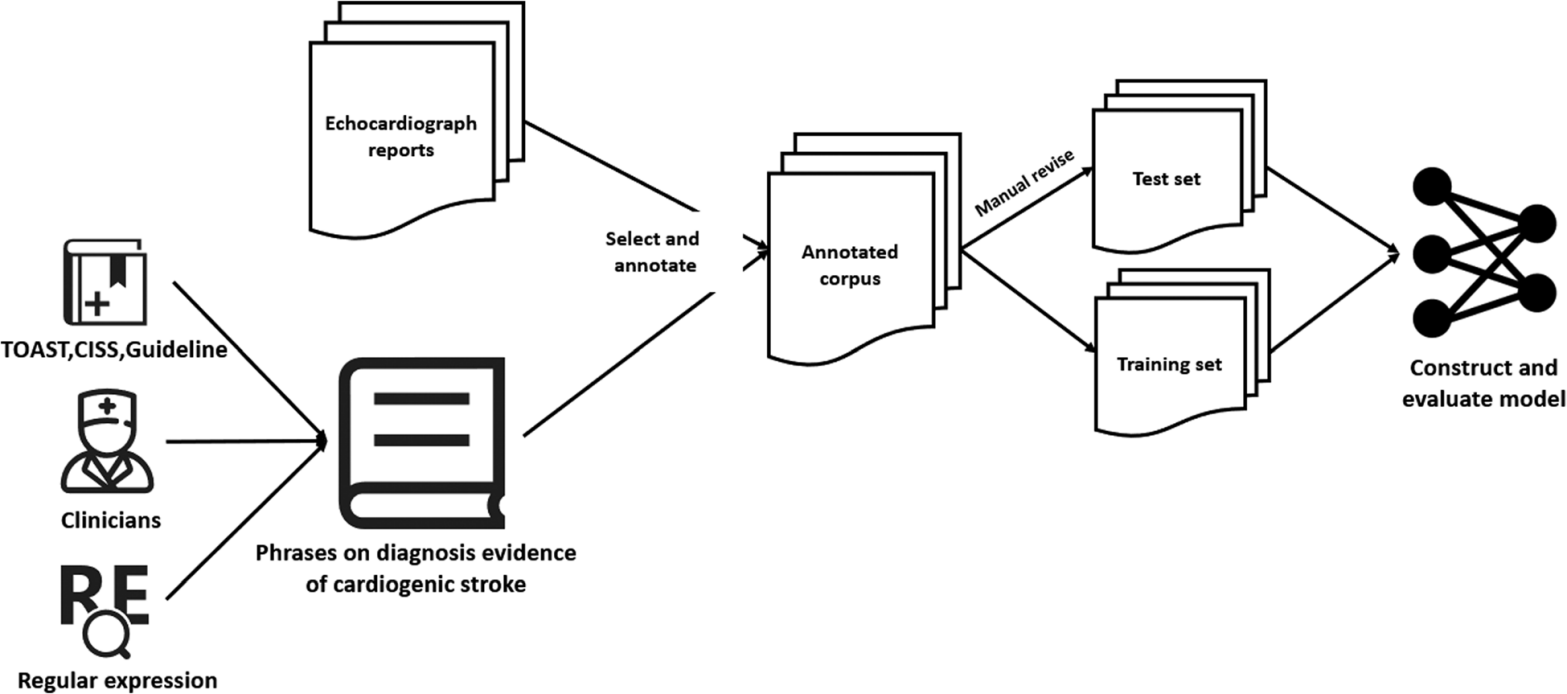
The workflow of this research

Figure 1 shows the workflow of this research. First of all, we summarized 149 phrases on diagnosis evidence of cardiogenic stroke through reviewing the relevant literature and extended using regular expressions in corporation with clinicians. Then we mapped the echocardiograph reports to the phrases to obtain the annotated corpus. Meanwhile, we selected the 11 most frequent diagnosis evidence of cardiogenic stroke from the corpus as entities for further research. Then the annotated corpus was divided into a training set and a testing set to construct and validate the identification model of diagnosis evidence. Besides, the testing set is further revised by clinicians as a gold standard. The study and data use were approved by the Human Research Ethics Committees of Beijing Tiantan Hospital, Capital Medical University, Beijing, China.

### Dataset

**Fig. 2:**
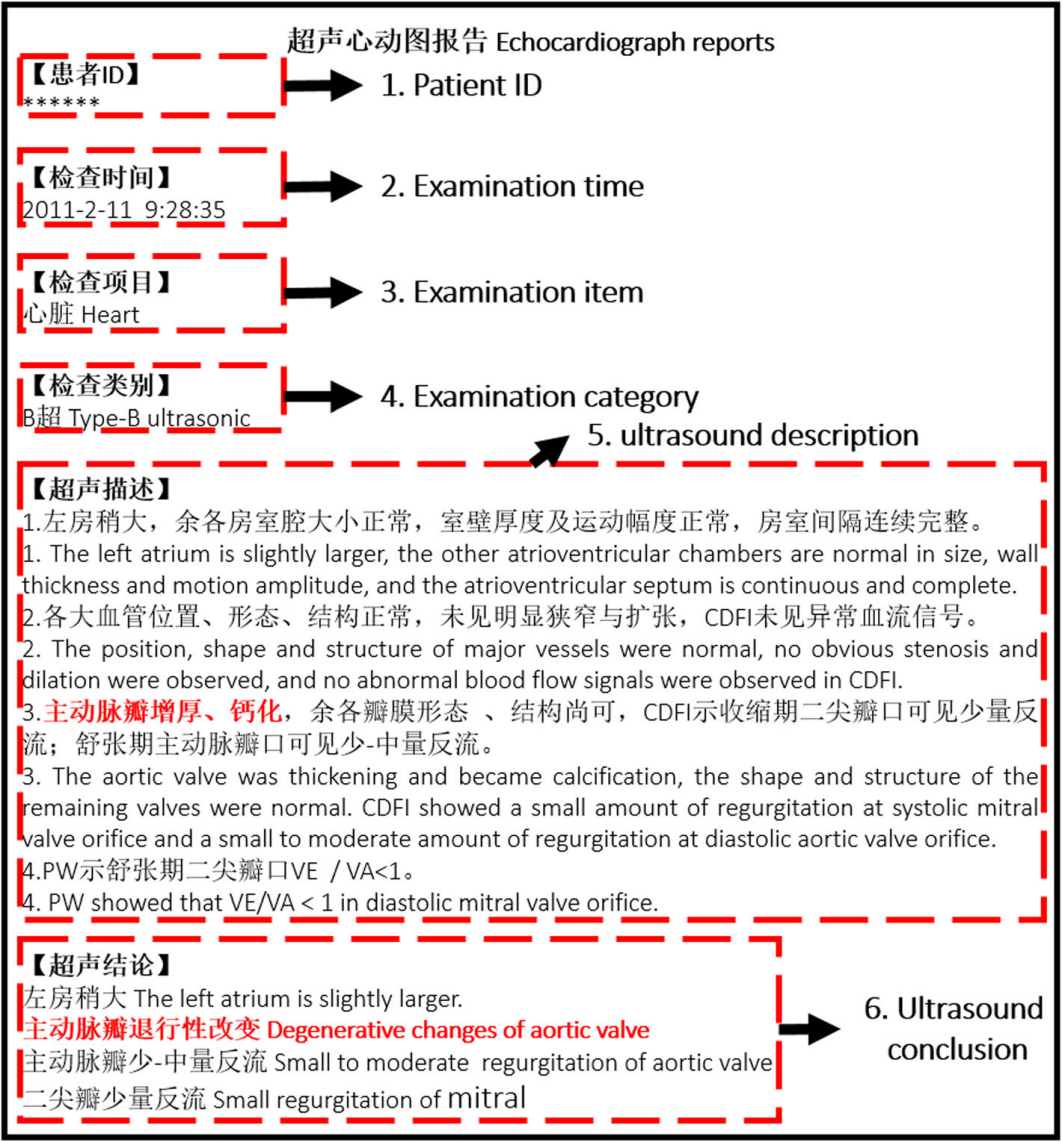
An example of transthoracic echocardiography report

**Fig. 3:**
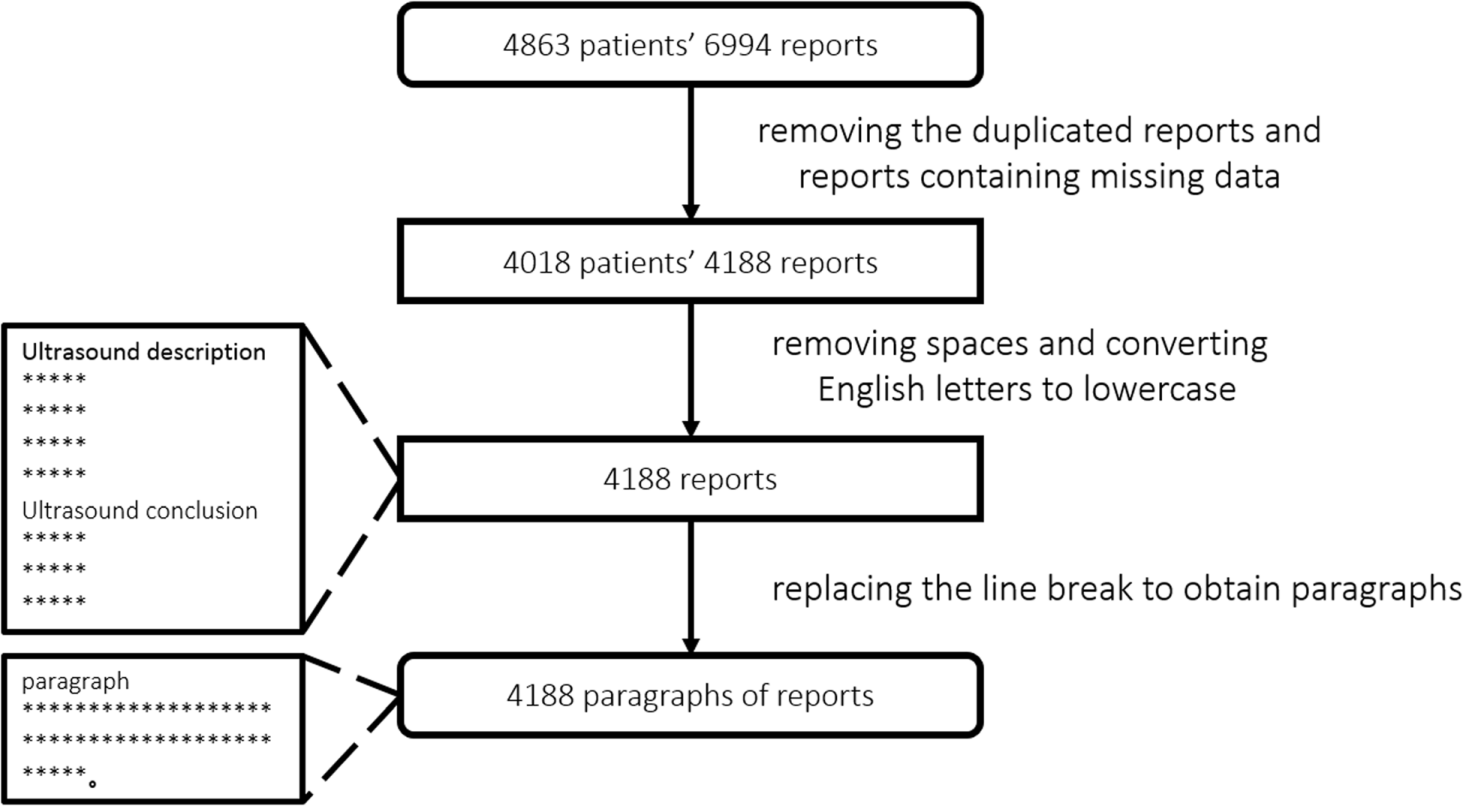
The data preprocessing

A total of 6994 transthoracic echocardiography reports from 2009 to 2018 were obtained from the electronic medical record system in Beijing Tiantan Hospital, Capital Medical University. After removing the duplicated reports and reports containing missing data, we obtained 4188 transthoracic echocardiography reports from 4018 patients. As shown in Fig. 2, a report contains six parts, including patient ID, examination time, examination item, examination category, ultrasound description, and ultrasound conclusion, the red font in report are diagnosis evidence of cardiogenic stroke. We preprocess the echocardiography reports, including removing spaces and converting English letters to lowercase. After observation, we found that ultrasound description and ultrasound conclusion is divided by line break, and each line is a sentence or phrase. After a preliminary statistical analysis of 4188 ultrasound conclusions, a total of 991 ultrasound conclusion phrases are obtained, we observed that because the doctor’s words are not standardized, there will be a number of phrases with the same meaning, for example, “主动脉瓣退行性改变” (aortic valve degeneration) and “主动脉瓣退行性变” (aortic valve degeneration), which make it difficult to identify diagnosis evidence of cardiogenic stroke from echocardiography reports. There is an average of 4 ultrasound description sentences (4 lines) in each echocardiography report, and each ultrasound description sentence has an average of 34 characters. On the other hand, there is an average of 3 ultrasound conclusion phrases (3 lines) in each echocardiography report, and each ultrasound conclusion phrase has an average of 8 characters. Then we replace the line break of ultrasound conclusion with comma, which converts ultrasound conclusion into one line and forms a sentence. At last we got a paragraph with an average of 177 Chinese characters from each report including ultrasound description sentences and ultrasound conclusion sentence without line break. The data preprocessing is shown in Fig. 3.

### Constructing vocabulary and annotating corpus

By referred to trial of org 10,172 in acute stroke treatment (TOAST) [25], Chinese ischemic stroke subclassification (CISS) [11], guidelines for the use of echocardiography in the evaluation of a cardiac source of embolism [26], and consulting neurologist, we got 20 diagnosis evidences of cardiogenic stroke that can be examined by transthoracic echocardiography, as shown in Table 1. Then two ultrasound clinicians were consulted about the frequently used-phrases on diagnosis evidence of cardiogenic stroke in the echocardiography reports, and we got 27 phrases on 20 diagnosis evidences of cardiogenic stroke, as shown in the second column of Table 1. After that we used the regular expression to expand these phrases, such as “二尖瓣狭窄”(mitral stenosis), a diagnosis evidence of cardiogenic, we used the regular expression “二尖瓣 [\u4e00-\u9fa5] * 狭窄” to match the phrases “二尖瓣轻度狭窄”, “二尖瓣中度狭窄” and “二尖瓣重度狭窄”. In the regular expression, the “[\u4e00-\u9fa5]” indicates matching Chinese characters, and after using the regular expression the extended phrases size reaches 55, which is shown in the third column of Table 1. At the same time, we observed that due to the writing habits of sonographers, aortic valve and mitral valve often formed compound diagnosis evidence of cardiogenic stroke, such as “主动脉瓣,二尖瓣钙化”(aortic and mitral valve calcification), which we added it to the phrases. In addition, according to the suggestion of sonographers and neurologists, we added “cardiac enlargement” (心脏增大), “ventricular wall hypokinesis” (室壁运动减弱) and “decrease of systolic heart function” (心脏收缩功能减弱) as potential diagnosis evidences of cardiogenic stroke into the phrases, and also extended them with the regular expressions, because these three phrases were closely related to diagnosis evidences of cardiogenic stroke, and they were also common in echocardiograph reports. The size of phrases reached 149 after expanding of these three potential diagnosis evidences of cardiogenic stroke and a compound diagnosis evidences of cardiogenic stroke.

**Table 1:** The diagnosis evidences of cardiogenic stroke

Diagnosis evidences of cardiogenic stroke	Phrases summarized by a clinicians	Phrases extended by Regular expressions
左心房/左心室附壁血栓 (left atrium / left ventricular mural thrombus)	左心房附壁血栓、左心室附壁血栓 (left atrium / left ventricular mural thrombus)	左心房附壁血栓、左心室附壁血栓 (left atrium / left ventricular mural thrombus)
心肌梗死 (myocardial infarction)	心肌梗死、节段性室壁运动异常 (myocardial infarction, regional wall motion abnormality)	心肌梗死、心梗、节段性室壁运动异常、节段性左室壁运动异常 (myocardial infarction, regional wall motion abnormality)
左心室室壁瘤 (left ventricular aneurysm)	室壁瘤 (vntricular aneurysm)	室壁瘤 (vntricular aneurysm)
扩张型心肌病 (dilated cardiomyopathy)	扩张型心肌病 (dilated cardiomyopathy)	扩张型心肌病、扩张性心肌病 (dilated cardiomyopathy)
人工瓣膜及心内辅助装置 (prosthetic valves and devices)	植入术、置换术 (implantation, replacement)	植入术、置换术、置入术、置换 (implantation, replacement)
瓣膜性心内膜炎 (valvular endocarditis)	感染性心内膜炎 (infective endocarditis)	感染性心内膜炎 (infective endocarditis)
心内肿物 (intracardiac mass)	肿物、团块、回声团 (mass, lump, echo group)	肿物、团块、回声团 (mass, lump, echo group)
粘液瘤 (myxoma)	粘液瘤 (myxoma)	粘液瘤 (myxoma)
乳头状弹力纤维瘤 (papilla elastic fibroma)	纤维瘤 (fibroma)	纤维瘤 (fibroma)
射血分数< 35% (ejection fraction< 35%)	射血分数低 (low ejection fraction)	射血分数低 (low ejection fraction)
左心室尖运动障碍 (left ventricular apex dyskinesia)	心尖运动减弱 (apex motion weakening)	心尖运动减弱、心尖运动略减弱 (apex motion weakening)
自显影 (spontaneous echocardiographic contras)	自显影 (spontaneous echocardiographic contras)	自显影 (spontaneous echocardiographic contras)
二尖瓣狭窄 (mitral stenosis)	二尖瓣狭窄 (mitral stenosis)	二尖瓣狭窄、二尖瓣轻度狭窄、 二尖瓣中度狭窄、二尖瓣重度狭窄 (mild, moderate, severe mitral stenosis)
二尖瓣脱垂 (mitral valve prolapse)	二尖瓣脱垂 (mitral valve prolapse)	二尖瓣脱垂、二尖瓣轻度脱垂、 二尖瓣前叶脱垂、二尖瓣后叶脱垂 (mitral valve prolapse)
二尖瓣钙化 (mitral valve calcification)	二尖瓣钙化、二尖瓣退行性改变 (mitral valve calcification, mitral valve degeneration)	二尖瓣钙化、二尖瓣退行性改变、二尖瓣退行性变、 … … (mitral valve calcification, mitral valve degeneration, … …)
主动脉瓣钙化 (aortic valve calcification)	主动脉瓣钙化、主动脉瓣退行性改变 (aortic valve calcification, aortic valve degeneration)	主动脉瓣钙化、主动脉瓣退行性改变、主动脉瓣退行性变、 … … (aortic valve calcification, aortic valve degeneration, … …)
巨大Lambl’s赘生物 (giant Lambl’s excrescences)	巨大Lambl’s赘生物 (giant Lambl’s excrescences)	巨大Lambl’s赘生物 (giant Lambl’s excrescences)
房间隔瘤 (atrial septal aneurysm)	房间隔瘤 (atrial septal aneurysm)	房间隔瘤 (atrial septal aneurysm)
房间隔缺损 (atrial septal defect)	房间隔缺损 (atrial septal defect)	房间隔缺损 (atrial septal defect)
充血性心力衰竭 (congestive heart-failure)	充血性心力衰竭 (congestive heart-failure)	充血性心力衰竭 (congestive heart-failure)

We used the forward maximum matching algorithm to map the phrases and paragraph of echocardiograph reports. The 10 of most common diagnosis evidences of cardiogenic stroke and potential diagnosis evidence of cardiogenic stroke were “心脏增大” (cardiac enlargement), “主动脉瓣钙化” (aortic valve calcification), “心肌梗死” (myocardial infarction), “二尖瓣钙化” (mitral valve calcification), “室壁运动减弱” (ventricular wall hypokinesis), “心脏收缩功能减弱” (decrease of systolic heart function), “人工瓣膜及心内辅助装置” (prosthetic valves and devices), “二尖瓣狭窄” (mitral stenosis), “左室附壁血栓” (left ventricular aneurysm) and “二尖瓣脱垂” (mitral valve prolapse). We selected these ten diagnosis evidences of cardiogenic stroke and a compound diagnosis evidence of cardiogenic stroke “主动脉瓣、二尖瓣钙化” (aortic and mitral valve calcification) for further study. The diagnosis evidences of cardiogenic mentioned later in this paper also refer to the selected diagnosis evidences of cardiogenic stroke, potential diagnosis evidences of cardiogenic stroke and compound diagnosis evidence of cardiogenic stroke.

**Fig. 4:**
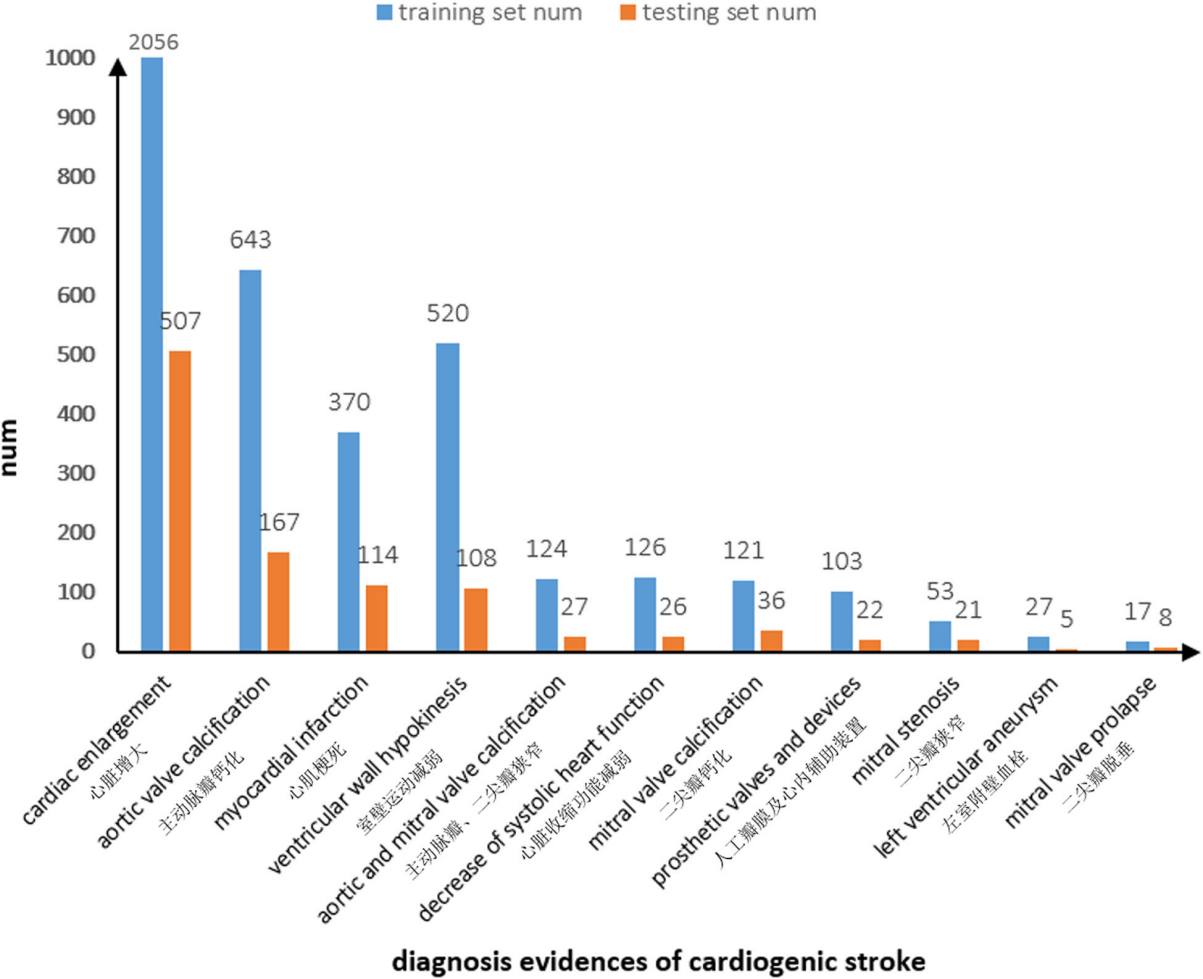
The distribution on diagnosis evidences of cardiogenic stroke in training set and test set

The extended phrases consist of selected diagnosis evidences of cardiogenic stroke was used to annotate the paragraph of reports with forward maximum matching algorithm. The annotation style is BIO. Then we divided the annotated of 4188 echocardiograph reports into 3350 training sets and 838 testing sets according to the proportion of 8:2. The distribution on diagnosis evidences of cardiogenic stroke in the training set and the testing set is shown in Fig. 4.

We invited a neurologist to revise the extended phrases annotated testing set to obtain gold standard annotated data. Neurologist found some diagnosis evidences of cardiogenic stroke were not annotated by the phrases, indicating that once the phrases were not collected, they would not be able to be identified, on the other hands the phrases needed to be maintained, expanded which consumes manual labor and material resources. Because NER model based on deep learning has the ability to find new words [27], we then used the NER model based on deep learning to identify the diagnosis evidences of cardiogenic stroke.

### Model and evaluation criteria

**Fig. 5:**
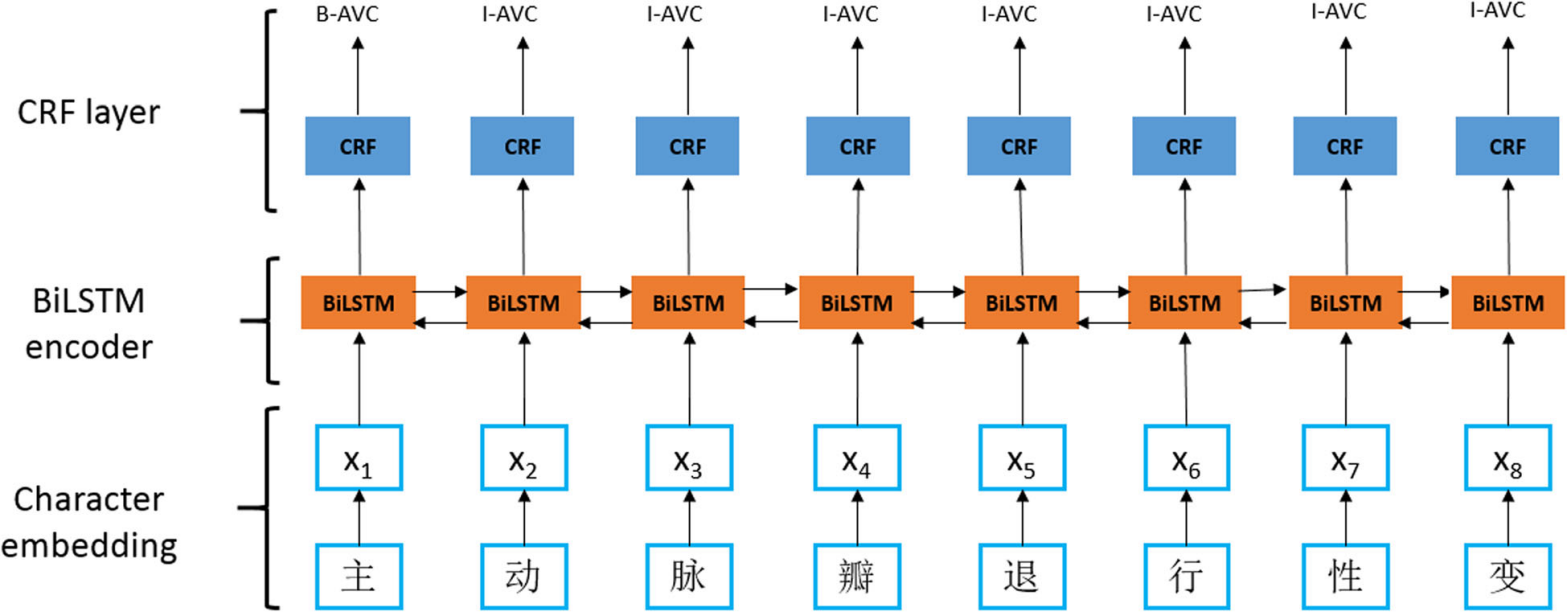
BiLSTM-CRF model

We selected Bi-directional Long Short-Term Memory-Conditional Random Field (BiLSTM-CRF) model [28] based on character vector to identify diagnosis evidences of cardiogenic, and the model structure is shown in Fig. 5. The BiLSTM-CRF model based on character vector is composed of three layers:

The first layer is the character embedding layer, and each character is corresponding to the character vector. In this paper, the word2vec model [29] is used to pre-train the character vector and we implemented it using the open source of gensim package. The training corpus consists of 4188 ultrasound descriptions and ultrasound conclusions, the parameter setting is 100 dimension of character vector and window is 5.

The second layer is a bidirectional LSTM layer, which automatically extracts the sentence features. The character vector sequence *x* = ( x_1_, x_2_, …, x_n_) is used as input, and then the sentence features are automatically extracted by bidirectional LSTM recorded as matrix P = ( p_1_, p_2_, …, p_n_) ∈ R^*n* × *k*^, k is the number of labels (in this study the k is 23). Each *p*_*i*_ dimension *p*_*ij*_ is regarded as the score of classifying character *x*_*i*_ to the jth label.

The third layer is the CRF layer, where sentence-level sequence tagging is carried out. The parameters of the CRF layer are a (k + 2) × (k + 2) matrix A. *A*_*ij*_ represents the transfer score from the ith label to the jth label. If we use a sequence of labels *y* = ( y_1_, y_2_, …, y_n_) which length is equal to the length of the sentence, the model scores when the label of the sentence x equals y is:

$$ \mathrm{score}\left(\mathrm{x},\mathrm{y}\right)=\sum \limits_{i=1}^n{P}_{i,{y}_i}+\sum \limits_{i=1}^{n+1}{A}_{y_{i-1},{y}_i} $$

For the evaluation criteria of the model, we use Precision, Recall and F1-Measure. The result set of the model prediction on the test set is recorded as *S* = ( s_1_, s_2_, …, s_m_), the gold standard set is recorded as *G* = ( g_1_, g_2_, …, g_n_). The set element is a diagnosis evidence of cardiogenic stroke, which is represented as a quadruples <d, *pos*_*b*_, *pos*_*e*_, c>, d is a echocardiography report, *pos*_*b*_ and *pos*_*e*_ are the starting and ending position of diagnosis evidences of cardiogenic stroke in report, and c is the label of diagnosis evidences of cardiogenic. We definite *s*_*i*_ ∈ *S* and *g*_*i*_ ∈ *G* is strictly equivalent only if:

1. $$ {s}_i\cdotp d={g}_j\cdotp d $$
2. $$ {s}_i\cdotp {pos}_b={g}_j\cdotp {pos}_b $$
3. $$ {s}_i\cdotp {pos}_e={g}_j\cdotp {pos}_e $$
4. $$ {s}_i\cdotp c={g}_j\cdotp c $$

Based on the above equivalent relationships, we define the intersection of the set S and set G is ⋂. The evaluation criteria indices are:

$$ \mathrm{P}=\frac{\left|S\cap G\right|}{\left|S\right|}\kern0.36em \mathrm{R}=\frac{\left|S\cap G\right|}{\left|G\right|}\kern0.36em \mathrm{F}1=\frac{2 PR}{P+R.} $$

## Results

**Fig. 6:**
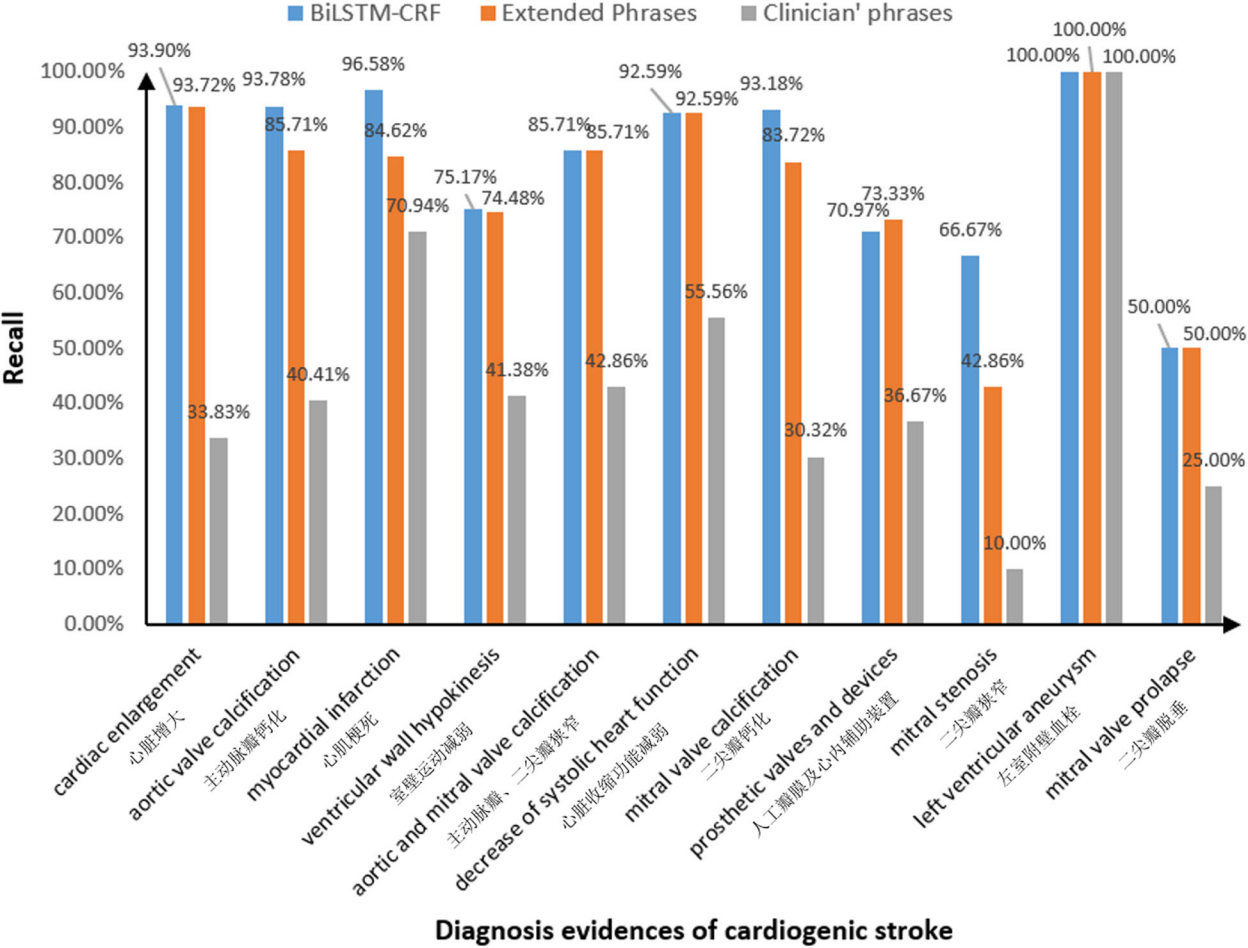
The Recall of three methods on the testing set

We set the learning rate at 0.0001 and the dropout at 0.5 to train the model. The number of hidden units in bidirectional LSTM-CRF is set to 100, and the optimizer is set to Adam. The overall precision, recall and F1-Measure of the BiLSTM-CRF model and phrases extended by the regular expressions annotating on the testing set are 98.03, 90.17, 93.94 and 99.21%, 86.21, 92.29% respectively, which indicates that overall performance of the BiLSTM-CRF model is better than phrases extended by the regular expression annotating on the aspect of Recall and F1-Measure.On the other hand, we pay attention to the ability finding new phrases of the BiLSTM-CRF model. Because from the definition of Recall, it can reflect whether the model identifies diagnosis evidences of cardiogenic stroke completely, and the higher the recall is, the more new phrases are identified when compared with the phrases annotating results. Therefore, we compared the recall of the two methods and using phrases summarized by clinicians annotating (not extended by regular expressions). From Fig. 6, we can find the BiLSTM-CRF model has the best performance, phrases extended by the regular expression is the second and phrases summarized by clinicians is the worst. Though in some categories the Recall of BiLSTM-CRF model is similar to phrases extended by the regular expression, in the categories of “主动脉瓣钙化” (aortic valve calcification), “心肌梗死” (myocardial infarction), “二尖瓣钙化” (mitral valve calcification) and “二尖瓣狭窄” (mitral stenosis), the Recall of the former is obviously higher than the latter, which indicates that the BiLSTM-CRF model identified more diagnosis evidences of cardiogenic stroke.

## Discussion

We analyze the error annotations of BiLSTM-CRF model predicted on the testing set, which can be divided into four types of errors, examples shown in Table 2. The first is the semantic dependence on long sentences does not work well, such as “二尖瓣前叶赘生物伴腱索断裂、瓣膜脱垂”(mitral valve prolapse), “主动脉瓣、二尖瓣瓣叶增厚、回声增强, 考虑退行性变”(aortic valve and mitral valve calcification) and so on, for this kind of long dependency in sentences, the performance of the model is poor. In the future, better models such as Transformer [30] can be used to extract sentence semantic features or increase the size of datasets maybe solving this problem. The second type of error is caused by similar grammatical structure of sentences, such as “二尖瓣位机械瓣” (mechanical mitral valve) is annotated as “prosthetic valves and devices”, but because it is followed by “轻度狭窄” (mild stenosis), the model annotates the latter “轻度狭窄” (mild stenosis) as “mitral stenosis”, because it similar with “二尖瓣轻度狭窄” (mild mitral stenosis), the model is easy to be confused at this situation, which leads to identification errors. The other is “阔瓣术后” (after wide flap surgery), which is not a diagnosis evidence of cardiogenic stroke, but because of “阔瓣术后” (after wide flap surgery) is similar to “植入术后” and “置换术后” (prosthetic valves and devices) at the aspect of grammatical structure, so it is identified as “prosthetic valves and devices” by the model, and another error is “三尖瓣脱垂” (tricuspid valve prolapse) is mistakenly identified as “mitral valve prolapse”, which is similar to “二尖瓣脱垂” (mitral valve prolapse). The third type of error is due to the phrase has big difference between those in training set. For example, “左室肥厚” (Left ventricular hypertrophy) is very different from “心脏增大” (cardiac enlargement), the same as “室壁运动僵直” (rigid wall ventricular wall motion) to “室壁运动减弱” (ventricular wall hypokinesis). The last error type is additional words at the end of phrase, such as “二尖瓣狭窄 (轻度)” (mitral stenosis (mild)), “轻度” (mild) is additional words at the end of “二尖瓣狭窄”(mitral stenosis), which is difficult for model to identify, especially, there are a lot of “二尖瓣狭窄”(mitral stenosis) instead of “二尖瓣狭窄 (轻度)” (mitral stenosis (mild)) in training set.

**Table 2:** Example of error in LSTM-CRF model prediction results on the testing set

Error type	Example	
	Gold standard	Prediction
Semantic dependence on long sentences	mitral valve prolapse: **二尖瓣前叶赘生物伴腱索断裂、瓣膜脱垂** Anterior leaflet vegetation of mitral valve with tendon rupture and valve prolapse	None
Similar grammatical structure of sentences	None	prosthetic valves and devices: **阔瓣术后** after wide flap surgery
Have big difference	ventricular wall hypokinesis: **室壁运动僵直** rigid wall ventricular wall motion	None
Additional words at the end of phrase	mitral stenosis: **二尖瓣狭窄** **(轻度)** mitral stenosis (mild)	mitral stenosis: **二尖瓣狭窄** mitral stenosis

Both the training set and the testing set are annotated by phrases, in which all data of the training set are annotated by phrases, which greatly saves the cost of manual annotation. When comparing the results of extended phrases annotation and gold standard on the test set, the F1 value of phrases annotation reaches 92.29%, which also shows the rationality of training BiLSTM-CRF model only with corpus annotated by phrases. We reflect on the feasible reasons for using phrases annotation in this study, because the phrases of diagnosis evidences of cardiogenic stroke are relatively fixed, the sonographers only add some qualifiers before, after and between the diagnosis evidences of cardiogenic stroke, or adopt abbreviations etc., after extending the phrases with regular expressions, most of the phrases can be covered. For this kind of identification task, the entities are relatively fixed, it is a wise choice to use phrases annotation, and it can save a lot of manual annotation cost. In addition, referring to the research of Komiya et al. when obtaining the gold standard annotation results, annotation was carried out with phrases at first, and then manual revise was carried out, which also saves the cost of all manual annotation.

We also analyzed the model’s ability to find new words. The BiLSTM-CRF model identified 17 new phrases, such as “二尖瓣中-重度狭窄”(mitral stenosis), “二尖瓣轻-中度狭窄”(mitral stenosis), “二瓣中-重度狭窄”(mitral stenosis), “主动脉瓣退行性病变”(aortic valve calcification), “主动脉退行性改变”(aortic valve calcification), “主动脉瓣增厚、钙化”(aortic valve calcification), “二尖瓣、主动脉瓣增厚, 退行性变”(aortic and mitral valve calcification), “节段性运动异常”(regional wall motion abnormality) and so on, among which “二瓣中-重度狭窄”(mitral stenosis), “节段性运动异常”(regional wall motion abnormality) and “主动脉退行性改变”(aortic valve calcification) are logically wrong, and not included in extended phrases,the correct writing is “二尖瓣中-重度狭窄”(mitral stenosis), “节段性室壁运动异常”(regional wall motion abnormality) and “主动脉瓣退行性改变”(aortic valve calcification), it shows that the BiLSTM-CRF model has the correction capability. Moreover because of the regular expression “二尖瓣[\u4e00-\u9fa5]*狭窄” can only match Chinese characters, the phrases contain “-” can’t identify by regular expression, which reveals that using the regular expansion to extend phrases requires continuous improvement of the rules to cover more phrases, and it often takes more manual labors and material resources to perfect the rules and extend the phrases.

## Conclusion

In this study, we identified the diagnosis evidences of cardiogenic stroke automatically. We explore a method of using phrases automatic annotation, and then training model to identify the diagnosis evidences of cardiogenic stroke. On the premise of only using phrases to annotate corpus, the identification model is trained and got a good performance. The precision, recall and F1-Measure of model on the testing set reaches 98.03, 90.17, 93.94% respectively, which shows the feasibility of this method. The constructed identification model of diagnosis evidences of cardiogenic stroke can automatically identify the common diagnosis evidences of cardiogenic stroke, which can assist neurologist to carry out diagnosis of cardiogenic stroke, and also provide support for the subsequent study of automatic etiological classification of acute ischemic stroke. However, there are also some limitations in this study: 1.The corpus is insufficiency, we can see that there are only 5 phrases at some type of diagnosis evidences of cardiogenic stroke in the testing set, which is not persuasive. We will expand the data set in future research to carry out a further evaluation. 2. Only a part of diagnosis evidences have been identified, and we selected the 11 most frequent diagnosis evidences of cardiogenic stroke to carry out our research. But we can’t ignore the rare diagnosis evidences of cardiogenic stroke in clinic, because if there is an omission, it may lead to a clinical accident. However, for the identification of rare diagnosis evidences of cardiogenic stroke, it is can’t be solved only by increasing the data set. We will continue to explore the identification of unfamiliar or rare diagnosis evidences of cardiogenic stroke in future research.

## Data Availability

Although we obtained permission from the institutional ethics committee to use the data, we did not obtain informed consent from patients to disclose medical history data. Therefore, the data can’t be available. But we will do our best to help other scholars who are interested in our research and hope to reproduce the results.

## Abbreviations

NER: Named Entity Recognition
NLP: Natural Language Processing
TOAST: Trial of Org 10,172 in Acute Stroke Treatment
CISS: Chinese Ischemic Stroke Subclassification
LAA: Large Artery Atherosclerosis
CS: Cardiogenic Stroke
PAD: Penetrating Artery Disease
OE: Other Etiology
UE: Undetermined Etiology
CRF: Conditional Random Field
RNN: Recurrent Neural Network
BiLSTM-CRF: Bi-directional Long Short-Term Memory-Conditional Random Field
